# Genetic effects of iron levels on liver injury and risk of liver diseases: A two-sample Mendelian randomization analysis

**DOI:** 10.3389/fnut.2022.964163

**Published:** 2022-09-16

**Authors:** Kai Wang, Fangkun Yang, Pengcheng Zhang, Yang Yang, Li Jiang

**Affiliations:** ^1^Department of Biochemistry and Molecular Biology, School of Basic Medical Sciences, Hangzhou Normal University, Hangzhou, China; ^2^Eye Center of the Second Affiliated Hospital, School of Medicine, Zhejiang University, Hangzhou, China; ^3^Zhejiang Provincial Key Lab of Ophthalmology, The Second Affiliated Hospital, School of Medicine, Zhejiang University, Hangzhou, China; ^4^Department of Cardiology, Ningbo First Hospital, School of Medicine, Zhejiang University, Ningbo, China; ^5^Department of Gastroenterology, First Affiliated Hospital, School of Medicine, Zhejiang University, Hangzhou, China; ^6^Department of Radiation Oncology, The Second Affiliated Hospital, Zhejiang University School of Medicine, Hangzhou, China

**Keywords:** iron, mendelian randomization, liver injury, non-alcoholic fatty liver disease, liver fibrosis/ cirrhosis

## Abstract

**Background and aims:**

Although iron homeostasis has been associated with liver function in many observational studies, the causality in this relationship remains unclear. By using Mendelian Randomization analyses, we aimed to evaluate the genetic effects of increased systemic iron levels on the risk of liver injury and various liver diseases. Moreover, in light of the sex-dependent iron regulation in human beings, we further estimated the sex-specific effect of iron levels in liver diseases.

**Methods:**

Independent single nucleotide polymorphisms associated with systemic iron status (including four indicators) at the genome-wide significance level from the Genetics of Iron Status (GIS) Consortium were selected as instrumental variables. Summary data for six liver function biomarkers and five liver diseases were obtained from the UK Biobank, the Estonian Biobank, the eMERGE network, and FinnGen consortium. Mendelian Randomization assessment of the effect of iron on liver function and liver diseases was conducted.

**Results:**

Genetically predicted iron levels were positively and significantly associated with an increased risk of different dimensions of liver injury. Furthermore, increased iron status posed hazardous effects on non-alcoholic fatty liver disease, alcoholic liver disease, and liver fibrosis/cirrhosis. Sex-stratified analyses indicated that the hepatoxic role of iron might exist in NAFLD and liver fibrosis/cirrhosis development among men. No significantly causal relationship was found between iron status and viral hepatitis.

**Conclusion:**

Our study adds to current knowledge on the genetic role of iron in the risk of liver injury and related liver diseases, which provides clinical and public health implications for liver disease prevention as iron status can be modified.

## Introduction

Iron is essential for many vital functions, however, there are no regulated means by which excess iron can be disposed of in humans. Therefore, whenever systemic iron exceeds its needs and storage capabilities are saturated, toxicity due to iron overload may arise (1). The liver plays a core role in systemic iron regulation. It is a major storage site for iron, taking up iron, and releasing iron back into the circulation (2). Additionally, the liver acts as a regulator in iron absorption and recycling for the organ to synthesize proteins to modulate iron homeostasis (3). Therefore, hepatic injury and dysfunction can disturb iron hemostasis. On the other hand, the presumption that iron-catalyzed oxidative injury plays a key role in the pathology of various forms of liver disease permeates the scientific and clinical literature (3). Iron is implicated in the pathogenesis of several human liver diseases, as hepatocellular and/or mesenchymal iron deposition was found in them. The type of liver siderosis (parenchymal, mesenchymal, or mixed) and its distribution throughout the lobule and the liver are useful means for suggesting its etiology (4). Accumulated observational studies have reported the association between iron overload and liver dysfunction (5–12). Nevertheless, confusion and conflict exist as the pathogenesis of various liver diseases is complicated. Moreover, for observational studies, it is difficult to distinguish causal and spurious associations due to problems of confounding and reverse causation.

Plasma concentration of liver enzymes (i.e., alanine aminotransferase [ALT], aspartate aminotransferase [AST], alkaline phosphatase [ALP], and gamma-glutamyl transferase [GGT], direct bilirubin [DBIL], and total bilirubin [TBIL]) are routinely measured clinical markers that represent different dimensions of liver function (13). Observational and laboratory studies suggested that systemic-iron regulation was associated with plasma concentrations of these enzymes (7, 10, 14). Iron overload is supposed to correlate with liver dysfunction, however, it is currently uncertain whether iron disorders contribute to, or result from liver dysfunction.

Genes are randomly allocated at conception, so that genetic effects on the exposure cannot be affected by classical confounding factors or reverse causation, as in the situation where the phenotype level is influenced by the presence of the disease (15). Mendelian Randomization (MR), where genetic variants that are strongly associated with a risk factor of interest are used to test its causal effect on an outcome, can help to distinguish causal effects from associations due to confounding or reverse causality (16). By studying the effect of iron status related to randomly allocated alleles, such an MR approach has previously been used in targeted analyses to investigate the effect of iron status on the risk of Parkinson's disease, coronary artery disease, stroke, and asthma (15, 17–19). However, so far, the causal effects of systemic iron status on liver function and related liver diseases are unclear.

In this work, we have extracted the largest available and updated datasets from UK Biobank to interrogate the potential effect of iron status (referred to serum iron, ferritin, transferrin, and transferrin saturation in a pattern consistent with an effect on systemic iron status), on liver function, proxied by multiple biomarkers (ALT, AST, ALP, GGT, DBIL, and TBIL), as well as on related liver diseases (including nonalcoholic fatty liver disease (NAFLD), alcoholic liver disease (ALD), viral hepatitis, liver fibrosis and/or cirrhosis and liver malignant neoplasm). Furthermore, we have also investigated whether systemic iron levels have a sex-specific effect on liver diseases since the morbidity of them are gender-dependent. Replication analyses in the largest liver disease genome-wide association study (GWAS) and reverse MR analysis were performed to comprehensively evaluate the iron-liver disease associations.

## Materials and methods

### Study design

**Figure 1:**
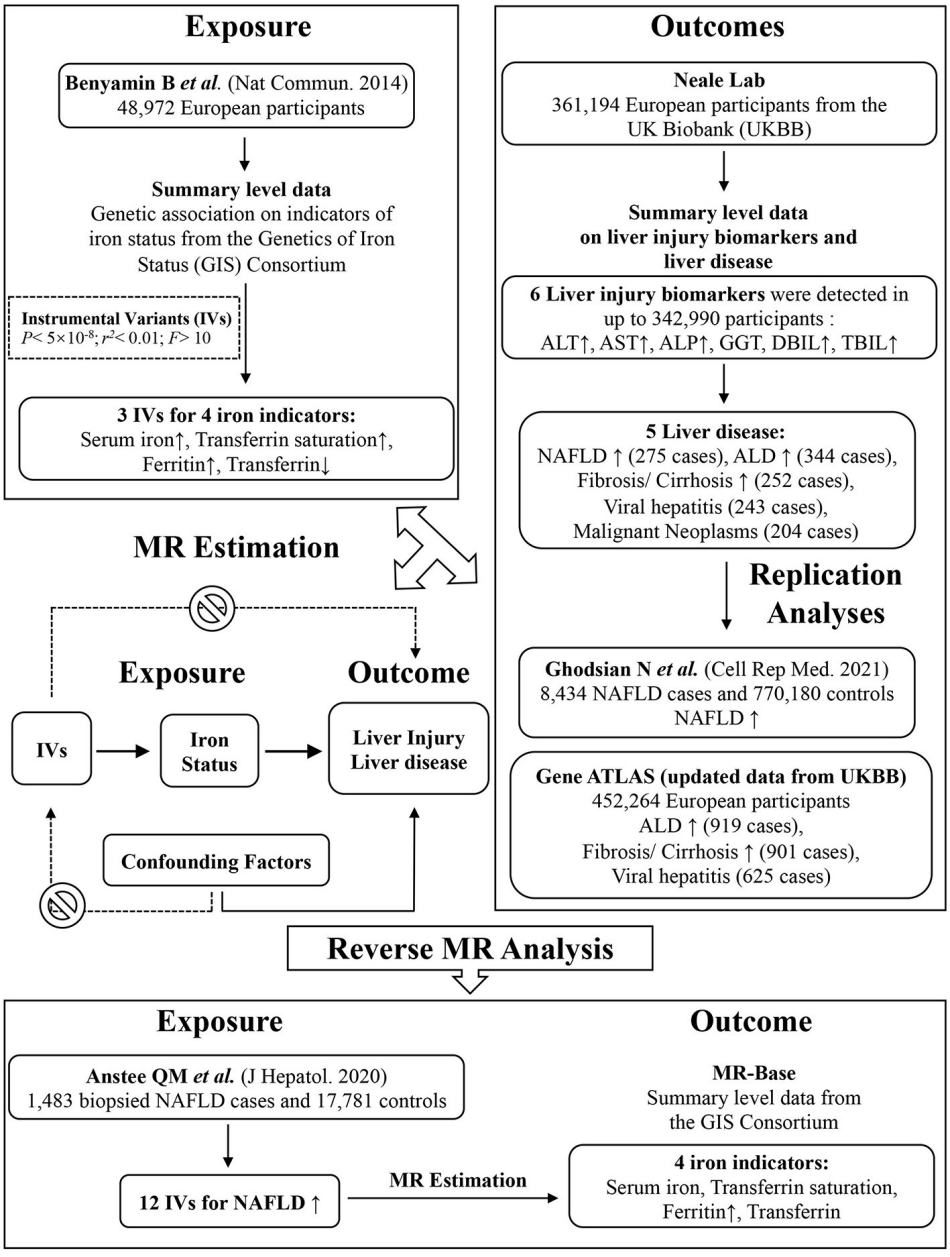
Graphical overview of the two-sample MR study design. Three SNPs, each of which has a genome-wide significant association with increased serum iron, increased ferritin, increased transferrin saturation and decreased transferrin levels, were used as instruments for systemic iron status. By using genetic instruments associated with these four iron status biomarkers, the MR approach can be used to estimate the causal effect of systemic iron status on the risk of liver function (biomarkers including ALP, ALT, AST, GGT, DBIL, TBIL) and liver disease (NAFLD, ALD, fibrosis and cirrhosis, viral hepatitis, malignant neoplasm). Replication and reverse MR analyses were performed in the largest available GWAS studies. MR, Mendelian randomization; SNP, single-nucleotide polymorphism.

This is a two-sample Mendelian Randomization study using summary statistics from two different studies to identify the causal effect of exposure on outcome. Appropriate patient consent and ethical approval were obtained in the original studies from which data for this work were obtained. The study design is depicted graphically in Figure 1.

### Genetic instruments

As genetic instruments for systemic iron status, we selected single-nucleotide polymorphisms (SNPs) that related to 4 clinically measured biomarkers. Increased systemic iron status is associated with increased serum iron, ferritin, and transferrin saturation, as well as decreasing transferrin levels (20). A GWAS performed by the Genetics of Iron Status (GIS) Consortium on 48,972 European subjects identified 3 such SNPs associated with all 4 biomarkers at genome-wide significance (*P* < 5 × 10^−8^): rs1800562 and rs1799945 in the hemochromatosis (*HFE*) gene and rs855791 in the transmembrane protease serine 6 (*TMPRSS6*) gene (21), with low linkage disequilibrium (LD: *r*^2^ < 0.01) between the two SNPs in the *HFE* gene (21, 22). Supplementary Table 1 shows all iron status instrumental SNPs were strong instruments for MR analysis as measured by *F*-statistics > 10 (23).

We also performed a reverse MR analysis to estimate the effect of NAFLD on the alteration of iron status. The NAFLD GWAS study was recruited from clinics at several leading European tertiary liver centers involving 1,483 biopsied NAFLD cases and 17,781 controls (24). Twelve SNPs associated with NAFLD at the genome-wide significance and with low linkage disequilibrium were established as genetic instruments (Supplementary Table 2).

### Study outcomes

The outcomes of our MR analysis were liver function biomarkers and liver diseases. We obtained associations with outcomes using summary statistics from the UK Biobank, provided by Neale Lab (http://www.nealelab.is/uk-biobank/), in 361,194 European descent participants. The study was adjusted for age, age square, and 20 principal components in sex-specific analysis and additionally adjusted for sex, interactions of sex with age and age square in the overall analysis. For liver function biomarkers, we retrieved GWAS of untransformed variable type (natural unit) from Neale Lab. Among all the liver diseases examined by Neale Lab, we excluded those of self-reported conditions, unclear origin of liver diseases, unspecific classification (such as other inflammatory liver diseases, other diseases of liver), relative small case numbers and duplicates. Therefore, five out of eighteen classified liver diseases were included in our primary analysis. The population of patients with an established diagnosis of liver disease was characterized according to the International Statistical Classification of Diseases and Related Health Problems of the 10th revision (ICD-10). Liver fibrosis/cirrhosis was diagnosed according to ICD10: K74.0 (Hepatic fibrosis) and K74.2 (Hepatic fibrosis with hepatic sclerosis). NAFLD diagnosis was established from hospital records according to ICD10: K75.81, ICD10: K76.0, and ICD10: K76.9. ALD was defined according to ICD10: K70 and hepatitis ICD10: K73 (25). Supplementary Tables 3, 4 showed mean level and standard deviation (*SD*) of the included biomarkers, as well as the numbers of participants and cases in analyses. The summary data and phenotype information of liver enzymes and liver diseases can be accessed from https://docs.google.com/spreadsheets/d/1kvPoupSzsSFBNSztMzl04xMoSC3Kcx3CrjVf4yBmESU/edit?ts=5b5f17db#gid=227859291. The extracted information and web link to each GWAS of the outcomes were provided in the Supplementary Data Sheet.

The replication analysis for NAFLD was conducted in an updated GWAS meta-analysis of four cohorts: The Electronic Medical Records and Genomics (eMERGE) network, the UK Biobank, the Estonian Biobank, and FinnGen. This GWAS meta-analysis included 8,434 NAFLD cases and 770,180 controls, making it the largest genome-wide analysis for a clinical diagnosis of NAFLD (24). In addition, we also performed replicate analysis in the most updated publicly available dataset of UK Biobank provided by Gene ATLAS (http://geneatlas.roslin.ed.ac.uk) (26). In 452,264 European descent participants, 901 cases of liver fibrosis/cirrhosis, 919 cases of ALD, and 625 cases of viral hepatitis were identified.

In the reverse MR analysis, the outcomes were the four indicators of iron status including serum iron, ferritin, transferrin saturation, as well as transferrin levels. Summary-level data for these iron markers were obtained from the Genetics of Iron Status (GIS) Consortium (21). Data from the Consortium were extracted through the MR-Base platform (27).

### Statistical analysis

To test the hypothesis that genetic instruments affect liver function and liver diseases by affecting iron levels, we utilized MR analysis methods. Wald estimates for each SNP were calculated as the ratio of genetic association on outcomes and genetic association on iron status (28). For liver biomarker, the effect estimate was beta of standardized liver biomarker level (*SD*). For liver disease, the effect estimate was odds ratio. Genetically determined 1-*SD* increase for iron indicators (or decrease for transferrin levels) were associated with an increase of beta (*SD*) for liver biomarkers or odds ratio for liver diseases. Standard errors were calculated using the Delta method (29). Then we performed fixed-effect inverse-variance weighted (IVW) meta-analysis for all 3 instrument SNPs to derive the overall MR estimate (23). A threshold of *P* < 0.05 was used to determine statistical significance.

For sensitivity analyses, we tested our findings in an updated dataset (Gene ATLAS) which included a larger sample size and more cases to evaluate whether the results were affected markedly by sample size. To further investigate the potential bias of the findings due to possible pleiotropy, we also used the weighted median and MR-Egger methods to confirm findings from our main analyses (30, 31). Specifically, the weighted median method is robust to invalid instruments and able to provide consistent estimation even when up to 50% of the weight is from invalid SNPs (30). The MR-Egger method may provide correct estimates as long as the instrument strength independent of direct effect assumption is satisfied. A non-null MR-Egger intercept suggests that the IVW estimate is invalid (31). Furthermore, we checked for secondary phenotypes associated with the selected instruments in two comprehensive curated genotype to phenotype cross-references, i.e., Ensembl (http://www.ensembl.org/index.html) and PhenoScanner (www.phenoscanner.medschl.cam.ac.uk). Statistical power was estimated by using an online tool (https://shiny.cnsgenomics.com/mRnd/) (32).

All statistical analyses were conducted using the “Mendelian Randomization” package in the statistical program R version 3.6.1 (R Foundation for Statistical Computing, Vienna, Austria).

## Results

### Genetic instruments for systemic iron status

We identified 3 SNPs associated with systemic iron status from the GIS meta-analysis. The effect on iron levels for each copy of the effect allele of instrument SNP expressed as the number of *SD* from the mean were listed in Supplementary Table 1. The *F*-statistics was high for all genetic instruments, as can be expected given the sample size of 48,972 individuals (21).

### Associations with liver biomarkers

Descriptive characteristics of the UK Biobank participants included in MR analyses, together with the number of participants and cases available for each outcome were provided in Supplementary Tables 3, 4.

**Figure 2:**
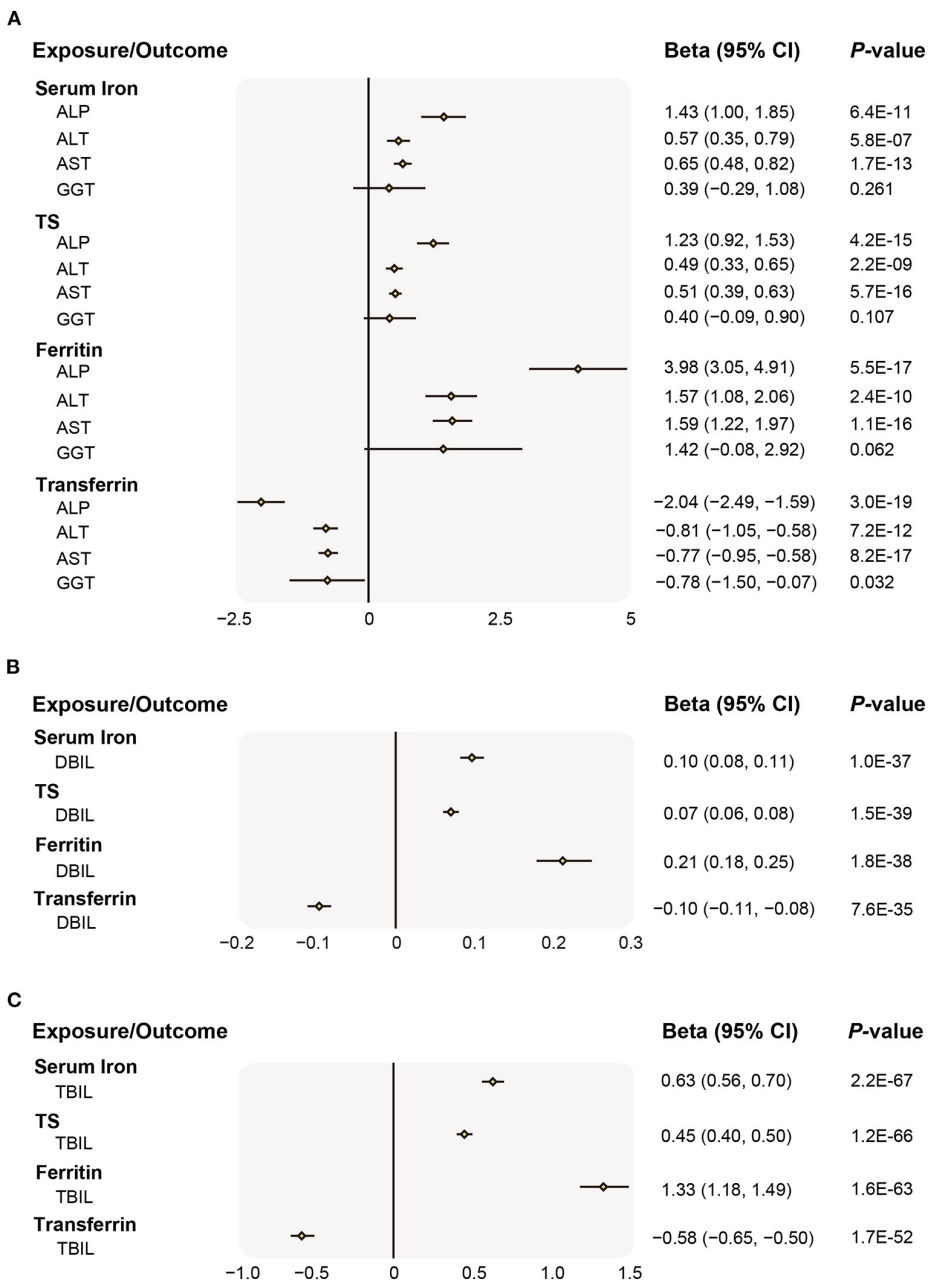
Associations of genetically preidicted iron status and liver biomarker. **(A)** Causal effects of iron status on ALP, ALT, AST, GGT; **(B)** Causal effects of iron status on DBIL; **(C)** Causal effects of iron status on TBIL. The beta (95% CI) of standardized liver biomarkers (ALP, ALT, AST, GGT, DBIL, and TBIL) per SD increase of iron status biomarkers were estimated using fixed-effect inverse-variance weighted meta-analysis. Beta, the Mendelian Randomization effect of continuous variable outcome; 95% CI, 95% confidence interval; ALP, alkaline phosphatase; ALT, alanine aminotransferase; AST, aspartate aminotransferase; GGT, gamma glutamyltransferase; DBIL, direct bilirubin; and TBIL, total bilirubin; SD, standard deviation.

Overall, genetic liability to higher iron status was associated with increased liver injury risk (indicated by ALP, ALT, AST, DBIL, and TBIL level), albeit the association with GGT was not statistically significant (Figures 2A–C). In main MR analyses (IVW), higher predisposition to genetically predicted serum iron (each increase in 1 *SD*) was strongly related to an increase of 1.43 SD of ALP (95% CI 1.00–1.85, *P* = 6.4 × 10^−11^), 0.57 *SD* of ALT (95% CI 0.35–0.79, *P* = 5.8 × 10^−7^), 0.65 *SD* of AST (95% CI 0.48–0.82, *P* = 1.7 × 10^−13^), 0.10 SD of DBIL (95% CI 0.08–0.11, *P* = 1.0 × 10^−37^), 0.63 *SD* of TBIL (95% CI 0.56–0.70, *P* = 2.2 × 10^−67^), but was not related to GGT (0.39, 95% CI −0.29 to 1.08, *P* = 0.261). Similarly, genetically determined increase (or decrease, for lower transferrin levels indicating higher iron status) in other iron status biomarkers were also significantly associated with the rise of ALP, ALT, AST, DBIL, and TBIL (Figures 2A–C). The associations were persistent in all sensitivity analyses including weighted median and MR-Egger method, though a few possible evidence of unbalanced horizontal pleiotropy was revealed by the MR-Egger method (Supplementary Table 5). A search of SNP-phenotype associations demonstrated that none of three SNPs were listed in either the Ensembl or PhenoScanner database as being strongly associated with traditional liver diseases risk factors.

### Associations with liver diseases

**Figure 3:**
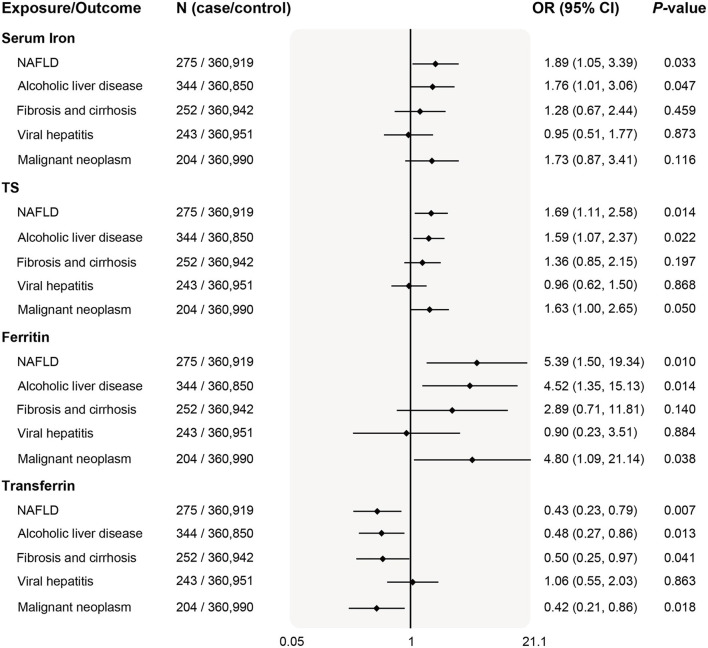
Associations of genetically predicted iron status and liver diseases. The OR (95% CI) of liver diseases (NAFLD, ALD, fibrosis and cirrhosis, viral hepatitis, and malignant neoplasm) per SD increase of iron status biomarkers were estimated using fixed-effect inverse-variance weighted meta-analysis. OR, odds ratio; 95% CI, 95% confidence interval; NAFLD, nonalcoholic fatty liver disease; ALD, alcoholic liver disease; SD, standard deviation.

Given the causal relationships between iron status and liver biomarkers, we further investigated the five classified liver diseases available in the Neale Lab. As shown in Figure 3, higher iron status was positively associated with risk of NAFLD (serum iron odds ratio 1.89, per SD unit increase; 95% CI 1.05–3.39, *P* = 0.033) and ALD (serum iron odds ratio 1.76, per *SD* unit increase; 95% CI 1.01–3.06, *P* = 0.047) based on the IVW estimates (*P* < 0.05 for all 4 iron status biomarkers). Similar results that deleterious effects of iron overload on the risk of NAFLD and ALD were also obtained for other instruments of iron status biomarkers (Figure 3).

In addition, serum transferrin was negatively related to liver fibrosis/cirrhosis (odds ratio 0.50, per *SD* unit increase; 95% CI 0.25–0.97, *P* = 0.041) and malignant neoplasm (odds ratio 0.42, per *SD* unit increase; 95% CI 0.21–0.86, *P* = 0.018). The positive associations remained consistent in the weighted median mode, albeit with wider CI in the MR-Egger method (Supplementary Table 6).

**Figure 4:**
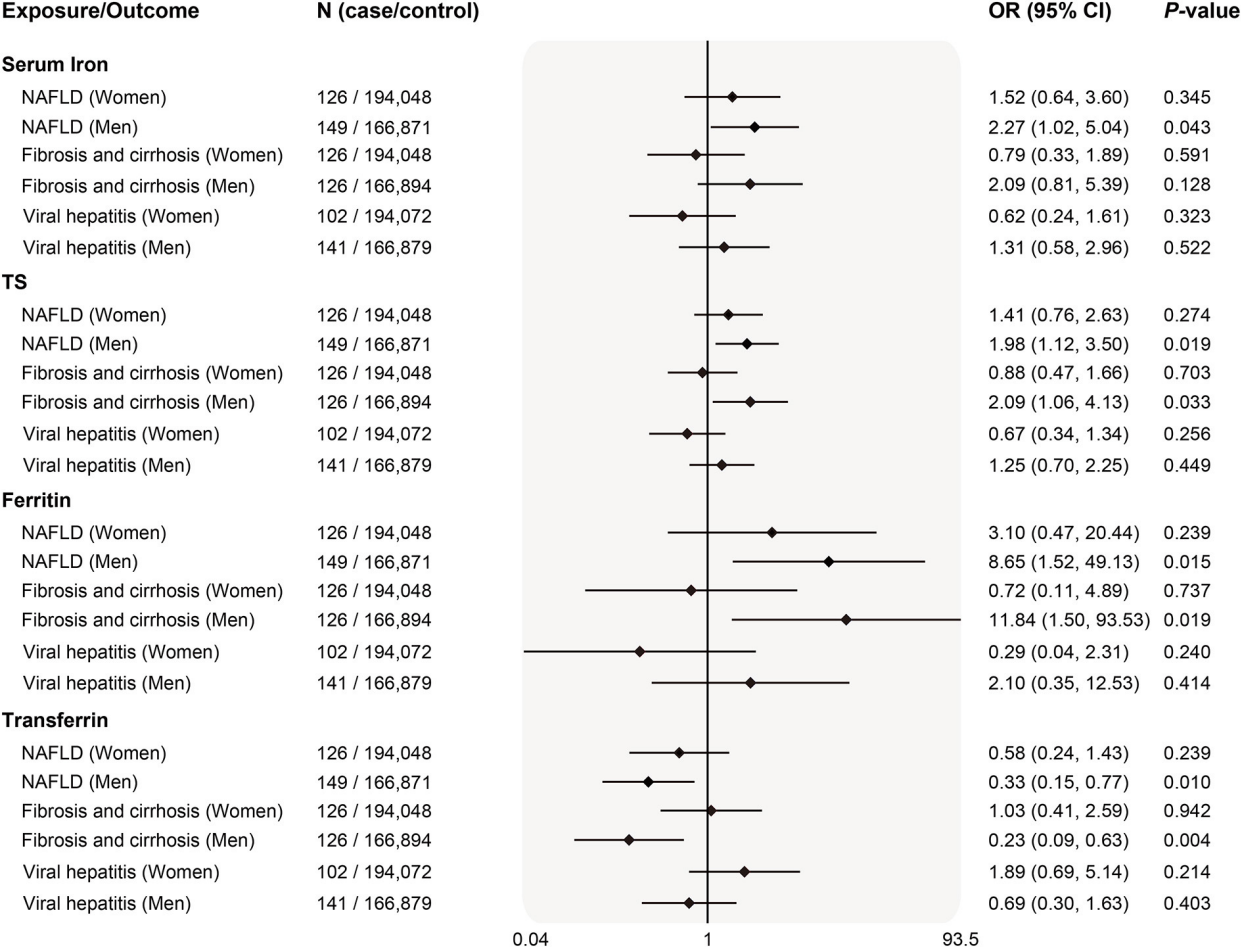
Sex-specific associations of genetically predicted iron status and liver diseases. The OR (95% CI) of liver diseases (NAFLD, fibrosis and cirrhosis and viral hepatitis) by sex per SD increase of iron status biomarkers were estimated using fixed-effect inverse-variance weighted meta-analysis. OR, odds ratio; 95% CI, 95% confidence interval; NAFLD, nonalcoholic fatty liver disease; SD, standard deviation.

Since the prevalence and severity of liver disease in many populations around the globe were gender-specific, we also examined sex differences in 3 diseases of which genetic summary data including both women and men were publicly available. Interestingly, genetically predicted systemic iron status was associated with higher risk of NAFLD in men (Figure 4). Similar causality was found for body systemic markers and liver fibrosis/cirrhosis risk in men after stratifying sex. There was no evidence for an association between iron and viral hepatitis both in women and men. Weighted median and MR-Egger estimates in the sensitivity analyses produced qualitatively consistent effects as the IVW estimates (Supplementary Table 7). Despite this, we observed that the power to demonstrate an insignificant relationship between iron status and liver diseases was limited (Supplementary Table 8).

### Replication analyses for iron-liver disease association

**Figure 5:**
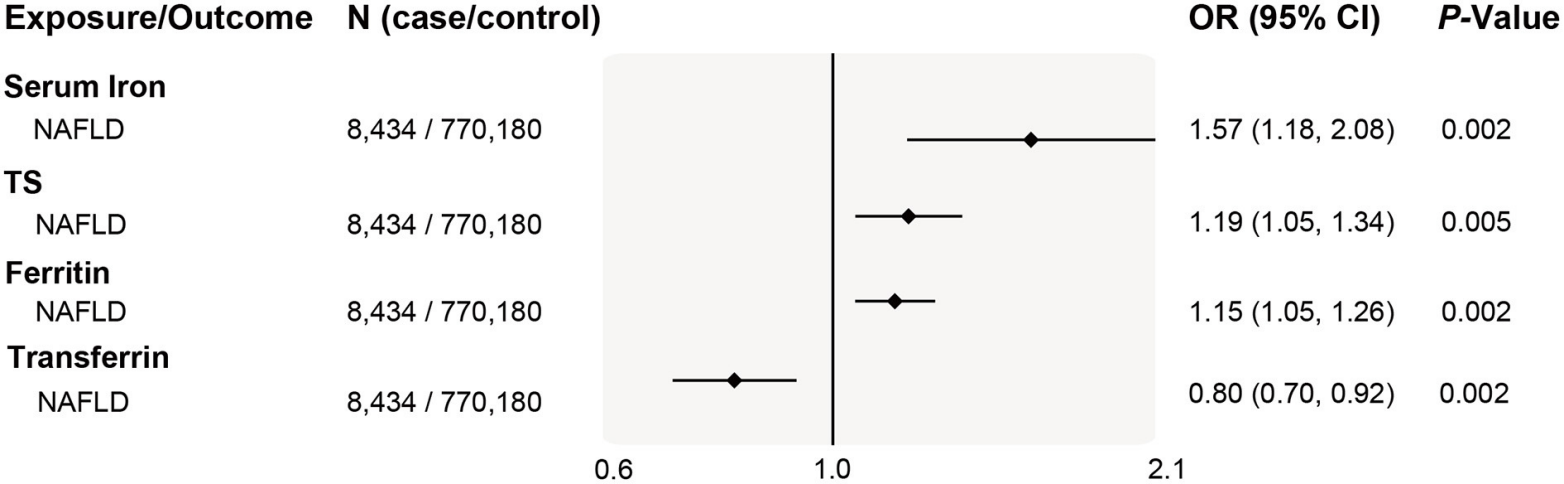
Replication of associations of genetically predicted iron status and NAFLD in the largest NAFLD GWAS study. The ORs (95% CI) of NAFLD per SD increase of iron status biomarkers were estimated using a fixed-effect inverse-variance weighted meta-analysis. OR, odds ratio; 95% CI, 95% confidence interval; NAFLD, nonalcoholic fatty liver disease; SD, standard deviation.

To comprehensively study the causality of iron- NAFLD association and minimize selection bias, we further performed an MR analysis in a recently reported NAFLD genome-wide meta-analysis. This study included 4 cohorts of electronic health record-documented NAFLD in participants of European ancestry (8,434 cases and 770,180 controls), representing the largest genome-wide analysis for a clinical diagnosis of NAFLD. Consistently, we found that genetic liability to higher systemic iron status was significantly and positively associated with NAFLD risk, odds ratios estimation of per SD increase in biomarker levels were 1.19 (95% CI 1.07–1.31, *P* = 5.3 × 10^−3^) for serum iron, 1.15 (95% CI 1.06–1.24, *P* = 2.3 × 10^−3^) for transferrin saturation, 1.57 (95% CI 1.29–1.85, *P* = 1.7 × 10^−3^) for ferritin, and 0.8 (95% CI 0.66–0.94, *P* = 1.9 × 10^−3^) for serum transferrin (Figure 5). The positive association between iron status and NAFLD risk was also replicated in both weighted median and MR-Egger estimations (Supplementary Table 9).

**Figure 6:**
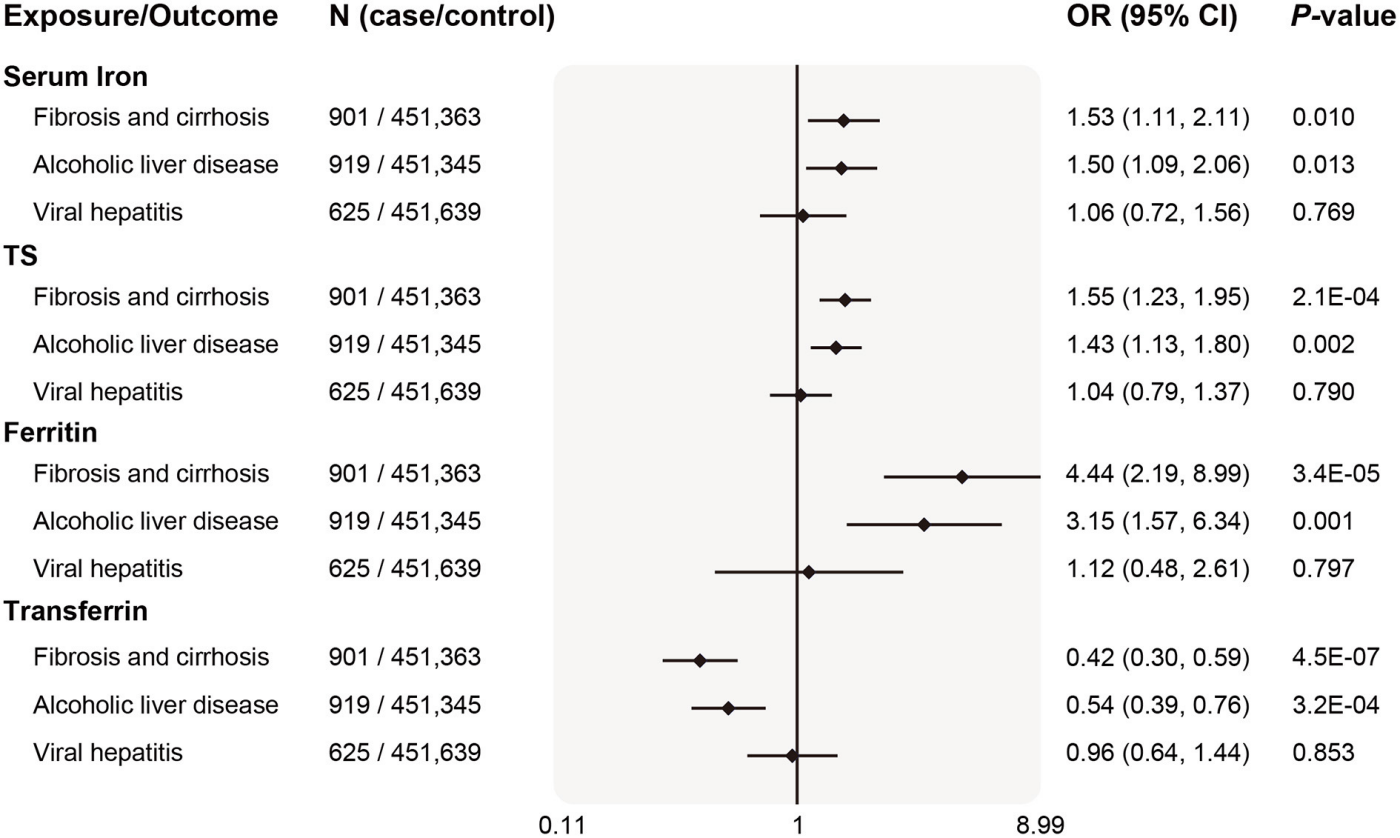
Replication of associations of genetically predicted iron status and liver diseases in the updated database. The ORs (95% CI) of liver diseases (ALD, fibrosis and cirrhosis, viral hepatitis) per SD increase of iron status biomarkers were estimated using fixed-effect inverse-variance weighted meta-analysis. OR, odds ratio; 95% CI, 95% confidence interval; ALD, alcoholic liver disease; SD, standard deviation.

For other liver diseases, after updating case number and sample size from Gene ATLAS (Figure 6), genetically determined higher systemic iron status (represented by all four biomarkers) became significantly associated with increased fibrosis and cirrhosis risk, odds ratios per *SD* increase in biomarker levels were 1.53 (95% CI 1.11–2.11, *P* = 0.010) for serum iron, 1.55 (95% CI 1.23–1.95, *P* = 2.1 × 10^−4^) for transferrin saturation, 4.44 (95% CI 2.19–8.99, *P* = 3.4 × 10^−5^) for ferritin, and 0.42 (95% CI 0.30–0.59, *P* = 4.5 × 10^−7^) for serum transferrin. In line with our primary analyses in Figure 3, systemic iron status was significantly associated with higher ALD risk, but had no causal effect on viral hepatitis.

### Reverse MR analyses

To assess the genetic effects of liver disease on iron levels, the MR estimates were performed in the opposite direction. By using the established IVs for NAFLD, our results showed no significant association for genetic liability to NAFLD on levels of iron status indicators, except for an association with ferritin level in the IVW mode (Supplementary Table 10).

## Discussion

Although multiple observational studies have reported the association between iron overload and liver dysfunction (7, 11, 14, 33), it is still unclear whether this association reflects causation. Our findings from the MR analyses showed strong evidence that genetic predisposition to higher systemic iron status was related to a higher risk of liver injury, which was reflected by increased blood concentration of ALT, AST, ALP, DBIL, and TBIL. In further analyses on liver diseases, our primary and replication results all showed that genetic liability to higher iron status was associated with increased risks of NAFLD, ALD, and liver fibrosis/cirrhosis. Moreover, our analyses stratified by sex indicated that genetically predicted iron status was associated with a higher risk of NAFLD and liver fibrosis/cirrhosis in men. Although serum iron wasn't found to be significantly associated with liver malignant neoplasm, other iron markers indicated the detrimental effect of iron on the progression of this severe disease. No strong evidence was found for a causal effect of genetically predicted iron status on viral hepatitis. In the reverse MR analyses, no significant genetic effects were observed for NAFLD progression on iron status.

Hereditary hemochromatosis (HH) is an iron overload disease and is the most common genetic condition in people of European descent (34). Observational evidence suggested that patients with HH were associated with a 4~11-fold risk of liver disease, including hepatocellular carcinoma, hepatitis C and nonalcoholic steatohepatitis (35). Other clinical studies also reported that HH-related iron overload can result in life-threatening clinical complications, most notably of which were severe liver diseases such as cirrhosis or hepatocellular carcinoma (HCC) (36, 37). Arguably, however, it remains confusing whether the risk of liver disease in HH homozygotes is attributable to disease penetrance, or from the independent hepatotoxic effect of iron. Epidemiological studies suffer from confounding and reverse causation, which is intrinsic to their observational nature so they can hardly provide conclusive evidence on the causality of an observed association. Thus, independent MR studies and large prospective trials using more accurate methods of diagnosing liver disease are needed to reach a conclusion on the crucial role of iron.

So far, this is the first MR study to examine the causal effect of predisposition to iron status on liver function. Previous observational studies observed an elevation of serum transaminases in most hemochromatosis patients and mouse models of iron-induced liver injury (38–40). Serum ferritin levels were also reported to associate with elevated liver function enzymes (7, 14). However, the intrinsic causality between iron status and liver function warrants to be clarified. Our results of increased systemic iron status cause elevated circulating liver injury biomarkers, including ALT, AST, ALP, DBIL, and TBIL, provide strong genetic evidence that excess iron could be a trigger for liver dysfunction.

The deleterious effect of higher iron levels on NAFLD and ALD found in our study may get support from previous observational and experimental studies. It is not uncommon that epidemiological studies point toward an association between high ferritin levels and the presence of NAFLD and steatohepatitis (7, 11, 14, 41, 42). As an indicator of systemic iron status, elevated serum ferritin concentration is an independent predictor of advanced hepatic fibrosis in patients with NAFLD (43). Accumulating evidence suggested that hepatic mitochondrial dysfunction might contribute to NAFLD development and severity, as occurs in experimental iron overload (44). Moreover, hepatic iron excess might contribute to the impairment of glucose homeostasis by influencing insulin signaling and metabolic control, reinforcing the idea of a possible role of iron in favoring NAFLD progression (45, 46). Sets of data suggested a correlation between systemic iron burden and the extent of liver damage in ALD patients (47, 48). The underlying pathogenic mechanisms of iron-promoted alcoholic steatohepatitis may be ascribed to the ability that alcohol and iron synergistically caused oxidative stress, stellate cell activation, and hepatic fibrogenesis, an exaggerated effect on liver disease progression (49). On the other hand, excess iron accumulation in Kupffer cells (KC) has emerged as a central event in hepatic toxicity in ALD (50), suggesting the dominant role of iron in the hepatic toxicity of ALD.

Studies have shown that the risk of cirrhosis is increased in iron-overloaded patients (51). Laboratory studies also reported that iron-generated oxyradicals and lipid peroxidation results in damage to hepatocellular organelles, which is thought to contribute to the development of hepatic fibrogenesis (52). It is highly necessary to use genetic variants to assess the genetic effect of iron exposure on liver cirrhosis. Our study supports the genetic association of increased iron levels with the risk of developing liver fibrosis/cirrhosis. The underlining mechanism of iron-triggered liver fibrosis/ cirrhosis progression could be explained by a newly identified, iron-dependent, and non-apoptotic cell death named ferroptosis (53). Previous studies reported that excess iron-induced liver ferroptosis played a key role in hepatic dysfunction and liver damage in different liver fibrosis/cirrhosis mouse models (54, 55), indicating the crucial effects of iron triggered ferroptosis in liver fibrosis/cirrhosis. Although epidemiologic studies have yielded mixed results for liver malignant neoplasm, there is an iron–cancer relationship among patients with HH, who are prone to develop liver cancer (33). However, we still emphasize that large prospective studies in selected groups of participants are needed to evaluate the independent pathogenic role of hepatic iron deposits in liver fibrosis/cirrhosis and malignant neoplasm.

In this study, we observed a sex-dependent casual association between iron status and NAFLD risk in men, similar sex-dimorphic result was also found for liver fibrosis/cirrhosis. These findings were corroborated by previous population-based studies which reported sex-specific prevalence of liver disease in HH patients, with the male gender increasing the estimated effect size for liver disease association (34). The prevalence of cirrhosis in the United States was reported to independently associated with the male sex (56). Males are also shown more susceptible to non-alcoholic fatty liver disease, non-alcoholic steatohepatitis, and liver fibrosis than females in humans or animals (57, 58). Previous observational studies suggested a significantly higher propensity of NAFLD among men with higher iron levels. The HH (H63D) mutation was reported to be significantly associated with male NAFLD patients, but not in female patients with NAFLD, suggesting that female patients with the H63D mutation might be protected from the development of NAFLD by iron loss through menstruation or pregnancy (59). In accordance with this, participants with higher dietary iron intake were subject to a higher prevalence of NAFLD in a dose-response relationship manner, moreover, after stratifying by gender, such association only remained in the male population (60). The observed sexual dimorphic associations between iron status with NAFLD and liver fibrosis/ cirrhosis progression may be explained by multiple pathways. It was established that iron accumulation occurs later in women, as serum ferritin levels in adult men were around 120 μg/L, while the values remained low in women at around 30 μg/L until menopause when ferritin levels increase to around 80 μg/L. This partially explained the stronger effect size of iron on liver disease among men. In addition, estradiol and its derivatives had a strong antioxidant capacity to suppress the generation of iron-induced reactive oxygen species and lipid peroxidation in the liver (61), which may prevent hepatocytes from oxidative damage, inflammation, and cell death.

Since randomized trials are very difficult to perform in the case of iron and liver disease, as investigating the long-term effect would require not only a very long follow-up but also a huge sample size, MR offers a cost-effective approach to derive well-powered causal effect estimates. However, the potential limitations of MR and this study deserve comment. In our study, genetic variants of iron status were used as IVs to explore the association between iron on liver function and liver disease based on 3 core MR assumptions: (1) The association between the genetic variants and the exposure is reliable, suggesting that the genetic IVs for the exposure should be selected at a genome-wide significance level (*P* < 5 × 10^−8^). In our MR study, we selected 3 SNPs strongly associated with all four iron status biomarkers (*P* < 5 × 10^−8^) that are broadly used in other MR studies (15, 17–19). The instrument strength was high for all of them, as shown by their *F*-statistic values. (2) The genetic variants should not be associated with any possible known confounders. (3) The genetic variants should affect the outcome only through the exposure. The second and third assumptions are known collectively as independence from pleiotropy. Pleiotropy is a potential source of bias specific to MR studies. In sensitivity analysis, we checked for unknown pleiotropy using the weighted median and MR-Egger methods and found little indication of pleiotropy. Investigation for SNP-associated secondary phenotypes using online databases also found no evidence of traditional liver disease risk factors, consistent with the assumption of no alternate causal pathways. Moreover, we also note that a Wald-type estimator for MR analysis of binary outcomes has been shown to induce bias, although typically of small magnitude (within 10%) (62). In addition, when interpreting our results, some concerns should be taken into consideration. Firstly, due to the low incidence of severe liver disease, the proportion of disease cases from UK Biobank was relatively small, which might reduce the effect size of the iron-disease estimate and result in statistically insufficient for MR analysis. However, our persistent results in primary and replication analyses showed a causal effect of iron overload on the enhanced risk of liver injury, providing evidence to reach the conclusion that increased iron is detrimental to liver function. Secondly, although our findings on the sex-specific analysis were supported by previous observational studies, it should be careful when interpreting the results as the statistical power was limited, suggesting that the insignificant associations might be due to a lack of power. Moreover, sex-specific IVs for iron status are not available. Therefore, further larger GWAS studies that explore the sex effects of iron are needed. Thirdly, due to not enough IVs being available for other liver diseases in current GWAS studies, the reverse MR analysis was conducted only for NAFLD patients. Moreover, a detailed fibrosis assessment was not available as the disease identification in the database has been generated from the ICD-10 codes. Further GWAS studies with a larger number and proportion of liver disease cases, and detailed information for disease characteristics, are warranted. Finally, to minimize bias from population stratification, we confined the study population to individuals of European ancestries, our findings may be limited when generalized to other populations with different ethnicities.

In conclusion, our study shows the genetic effects of increased iron levels on a higher risk of liver injury, and the development of NAFLD, ALD, and liver fibrosis/cirrhosis. In addition, sex-specific analyses found the hepatoxic role of iron in NAFLD and liver fibrosis/cirrhosis progression among men. These findings provide genetic evidence that disrupted iron metabolism may be a trigger in the pathogenesis of liver injury. Independent GWAS and large prospective studies are warranted to further validate our findings in other cohorts and ethnicities.

## Data availability statement

The original contributions presented in the study are included in the article/Supplementary material, further inquiries can be directed to the corresponding authors.

## Ethics statement

Ethical review and approval was not required for this human study as it was conducted using summary data obtained from a public database.

## Author contributions

LJ and KW: responsible for the writing of the original draft. LJ, KW, FY, YY, and PZ: reviewing and editing the manuscript and for acquisition, analysis, or interpretation of data. LJ, KW, and FY: conceptualization of the study, statistical analysis, and accessed the database and raw data. LJ: supervision. All authors contributed to the article and approved the submitted version.

## Funding

This study was supported by research grants from the National Natural Science Foundation of China (32000820 to LJ) and the China Postdoctoral Science Foundation (2022M712763 to KW).

## Conflict of interest

The authors declare that the research was conducted in the absence of any commercial or financial relationships that could be construed as a potential conflict of interest.

## Publisher's note

All claims expressed in this article are solely those of the authors and do not necessarily represent those of their affiliated organizations, or those of the publisher, the editors and the reviewers. Any product that may be evaluated in this article, or claim that may be made by its manufacturer, is not guaranteed or endorsed by the publisher.

## Abbreviations

GWAS: genome-wide association study
NAFLD: Nonalcoholic fatty liver disease
ALT: alanine aminotransferase
AST: aspartate aminotransferase
ALP: alkaline phosphatase
GGT: gamma-glutamyl transferase
DBIL: direct bilirubin
TBIL: total bilirubin
HFE: hemochromatosis protein
IVW: inverse variance weighted
MR: Mendelian Randomization
SNP: single-nucleotide polymorphism
TMPRSS6: transmembrane serine protease 6
GIS: Genetics of Iron Status
UKBB: UK Biobank
HH: Hereditary haemochromatosis
ALD: Alcoholic liver disease
HCC: hepatocellular carcinoma
KC: Kupffer cells
CHC: chronic hepatitis C
HBV: hepatitis B virus
NASH: non-alcoholic steatohepatitis
CLDs: chronic liver diseases
SD: standard deviation
OR: odds ratio
CI: confidence interval.
