# Supplementary material for: Genetic effects of iron levels on liver injury and risk of liver diseases: A two-sample Mendelian randomization analysis
Source: Front Nutr. 2022 Sep 16;9:964163. doi: 10.3389/fnut.2022.964163 (PMC9523310; doi:10.3389/fnut.2022.964163)
Supplement: Supplementary file 2 [file Data_Sheet_2.docx]

**Supplementary Materials**

**Causal Effects of Iron Levels on Liver Injury and Risk of Liver Diseases: A Two-sample Mendelian Randomization Analysis**

Kai Wang, Fangkun Yang, Pengcheng Zhang, Yang Yang, Li Jiang

**CONTENTS:**

**Table S1.** Association of genetic instruments with iron-status marker.

**Table S2**. Summary information for the SNPs of NAFLD for reverse MR analyses.

**Table S3.** Characteristics of participants from the UK Biobank by Neale Lab.

**Table S4.** The number of participants and cases in analyses from the UK Biobank.

**Table S5.** Associations between genetically predicted systemic iron status and liver biomarkers using weighted median and MR-Egger method.

**Table S6.** Associations between genetically predicted systemic iron status and liver diseases using weighted median and MR-Egger method.

**Table S7.** Sex-specific associations of genetically predicted iron status and liver diseases using weighted median and MR-Egger method.

**Table S8**. Statistical power for sex-specific associations in women.

**Table S9**. Weighted median and MR-Egger estimators of genetically predicted systemic iron status with NAFLD in the replication analysis.

**Table S10**. Associations of genetically predicted NAFLD with systemic iron status in reverse MR analyses.

**Table S1.** Association of genetic instruments with iron-status marker.

|  | | | | **Serum Iron (μmol/L)** | | | |  | **Transferrin Saturation (%)** | | | |  | **Ferritin (log, μg/L)** | | | |  | **Transferrin (g/L)** | | | |
| --- | --- | --- | --- | --- | --- | --- | --- | --- | --- | --- | --- | --- | --- | --- | --- | --- | --- | --- | --- | --- | --- | --- |
| **SNP** | **Gene** | **EA** | **EAF** | **R^2^** | **F** | ***β*** | **SE** |  | **R^2^** | **F** | ***β*** | **SE** |  | **R^2^** | **F** | ***β*** | **SE** |  | **R^2^** | **F** | ***β*** | **SE** |
| rs1800562 | *HFE* | A | 0.067 | 1.345 | 668 | 0.328 | 0.016 |  | 4.162 | 2127 | 0.577 | 0.016 |  | 0.520 | 256 | 0.204 | 0.016 |  | 2.869 | 1446 | -0.479 | 0.016 |
| rs1799945 | *HFE* | G | 0.15 | 0.911 | 450 | 0.189 | 0.01 |  | 1.361 | 676 | 0.231 | 0.01 |  | 0.108 | 53 | 0.065 | 0.01 |  | 0.331 | 163 | -0.114 | 0.01 |
| rs855791 | *TMPRSS6* | G | 0.554 | 1.619 | 806 | 0.181 | 0.007 |  | 1.784 | 889 | 0.19 | 0.008 |  | 0.149 | 73 | 0.055 | 0.007 |  | 0.096 | 47 | -0.044 | 0.007 |

Genetic instruments were selected based on the Genetics of Iron Status consortium study (N=48,972). SNP, single-nucleotide polymorphism; EA indicates effect allele; EAF, effect allele frequency; R^2^, percentage of the iron marker variation explained by the SNP; F, F statistic; HFE, gene encoding the hemochromatosis protein; TMPRSS6, gene encoding transmembrane serine protease 6.

**Table S2**. Summary information for the SNPs of NAFLD for reverse MR analyses.

| **SNP** | **Chr** | **Pos** | **Gene** | **Beta** | **Se** | **EA** | **NEA** | ***P-*Value** |
| --- | --- | --- | --- | --- | --- | --- | --- | --- |
| rs12077210 | 1 | 65894160 | LEPR | 0.39 | 0.07 | T | C | 5.62E-08 |
| rs1260326 | 2 | 27730940 | GCKR | 0.25 | 0.04 | T | CG | 1.06E-10 |
| rs1919127 | 2 | 27801493 | C2orf16 | 0.25 | 0.04 | C | T | 5.61E-10 |
| rs2068834 | 2 | 27839539 | ZNF512 | 0.26 | 0.04 | C | T | 8.49E-11 |
| rs9992651 | 4 | 88232510 | HSD17B13 | -0.3 | -0.05 | A | G | 2.78E-08 |
| rs13118664 | 4 | 88239609 | HSD17B13 | -0.3 | -0.05 | T | A | 1.41E-08 |
| rs139648192 | 8 | 39914918 | IDO2 | 0.43 | 0.08 | T | C | 5.20E-08 |
| rs58542926 | 19 | 19379549 | TM6SF2 | 0.48 | 0.07 | T | C | 2.05E-11 |
| rs8107974 | 19 | 19388500 | SUGP1 | 0.49 | 0.07 | T | A | 2.58E-12 |
| rs17216588 | 19 | 19664077 | CILP2 | 0.48 | 0.06 | T | C | 7.25E-14 |
| rs10500212 | 19 | 19723215 | PBX4 | 0.44 | 0.06 | T | C | 3.40E-12 |
| rs738409 | 22 | 44324727 | PNPLA3 | 0.6 | 0.04 | G | C | 1.45E-49 |

SNP, single nucleotide polymorphism; NAFLD, nonalcoholic fatty liver disease; Chr, Chromosome; Pos, Position; EA, Effect Allele; NEA, Non-Effect Allele.

**Table S3.** Characteristics of participants from the UK Biobank by Neale Lab.

| **Characteristics** | **Mean/N (Standard Deviation)** |
| --- | --- |
| No. (%) of women | 53.8 |
| Age (years) | 56.5 (8.1) |
| Body Mass Index (kg/m2) | 27.4 (4.8) |
| Alkaline phosphatase (U/L) | 83.7 (26.4) |
| Alanine aminotransferase (U/L) | 23.5 (14.1) |
| Aspartate aminotransferase (U/L) | 26.2 (10.6) |
| Gamma glutamyltransferase (U/L) | 37.3 (41.9) |
| Direct bilirubin (umol/L) | 1.8 (0.8) |
| Total bilirubin (umol/L) | 9.1 (4.4) |

**Table S4.** Number of participants and cases in analyses from the UK Biobank.

| **Analysis** | | **Participants number** | **Cases number** |
| --- | --- | --- | --- |
| Biomarkers | |  |  |
|  | Alkaline phosphatase | 344,292 | NA |
|  | Alanine aminotransferase | 344,136 | NA |
|  | Aspartate aminotransferase | 342,990 | NA |
|  | Gamma glutamyltransferase | 344,104 | NA |
|  | Direct bilirubin | 292,933 | NA |
|  | Total bilirubin | 342,829 | NA |
| Diseases from Neale Lab | |  |  |
|  | Nonalcoholic fatty liver disease | 361,194 | 275 |
|  | Alcoholic liver disease | 361,194 | 344 |
|  | Fibrosis and cirrhosis | 361,194 | 252 |
|  | Viral hepatitis | 361,194 | 243 |
|  | Malignant neoplasm | 361,194 | 204 |
| Diseases from Gene ATLAS | |  |  |
|  | Fibrosis and cirrhosis | 452,264 | 901 |
|  | Alcoholic liver disease | 452,264 | 919 |
|  | Viral hepatitis | 452,264 | 625 |

NA, Not Available.

**Table S5**. Associations between genetically predicted systemic iron status and liver biomarkers using weighted median and MR-Egger method.

| **Exposure** | **Outcome** | **Weighted median** | | | |  | **MR-Egger** | | | | |
| --- | --- | --- | --- | --- | --- | --- | --- | --- | --- | --- | --- |
|  |  | **Beta**^1^ | **95% CI** | | ***P*-value** |  | **Beta**^1^ | **95% CI** | | ***P*-value** | **Intercept *P*-value** |
| Serum Iron | ALP | 1.17 | 0.54 | 1.81 | 2.8E-04 |  | 7.23 | 3.13 | 11.34 | 5.6E-04 | 0.004 |
| TS | ALP | 1.28 | 0.90 | 1.66 | 3.1E-11 |  | 2.72 | 0.80 | 4.65 | 0.005 | 0.085 |
| Ferritin | ALP | 4.26 | 3.02 | 5.50 | 1.6E-11 |  | 7.13 | 2.84 | 11.41 | 0.001 | 0.085 |
| Transferrin | ALP | -2.11 | -2.62 | -1.60 | 4.5E-16 |  | -2.38 | -4.43 | -0.33 | 0.023 | 0.625 |
| Serum Iron | ALT | 0.36 | 0.01 | 0.72 | 0.046 |  | 2.77 | 1.87 | 3.66 | 1.3E-09 | 6.5E-07 |
| TS | ALT | 0.44 | 0.26 | 0.62 | 2.1E-06 |  | 1.06 | 0.71 | 1.40 | 1.7E-09 | 2.5E-04 |
| Ferritin | ALT | 1.51 | 0.93 | 2.09 | 3.8E-07 |  | 2.74 | 1.85 | 3.62 | 1.4E-09 | 0.002 |
| Transferrin | ALT | -0.84 | -1.08 | -0.59 | 1.7E-11 |  | -0.94 | -1.32 | -0.56 | 1.1E-06 | 0.326 |
| Serum Iron | AST | 0.37 | 0.11 | 0.64 | 0.006 |  | 2.05 | 1.36 | 2.74 | 6.4E-09 | 4.2E-05 |
| TS | AST | 0.49 | 0.35 | 0.63 | 3.8E-12 |  | 0.78 | 0.52 | 1.05 | 7.6E-09 | 0.023 |
| Ferritin | AST | 1.58 | 1.12 | 2.04 | 1.5E-11 |  | 2.03 | 1.34 | 2.71 | 6.6E-09 | 0.135 |
| Transferrin | AST | -0.75 | -0.94 | -0.56 | 7.7E-15 |  | -0.70 | -0.94 | -0.45 | 3.0E-08 | 0.412 |
| Serum Iron | GGT | 0.51 | -0.40 | 1.42 | 0.271 |  | 3.82 | -1.45 | 9.09 | 0.155 | 0.188 |
| TS | GGT | 0.58 | 0.01 | 1.15 | 0.048 |  | 1.40 | -0.80 | 3.60 | 0.213 | 0.317 |
| Ferritin | GGT | 1.94 | 0.25 | 3.63 | 0.024 |  | 3.74 | -1.59 | 9.07 | 0.169 | 0.307 |
| Transferrin | GGT | -1.07 | -1.83 | -0.31 | 0.006 |  | -1.18 | -3.33 | 0.96 | 0.279 | 0.577 |
| Serum Iron | DBIL | 0.10 | 0.08 | 0.13 | 1.4E-17 |  | 0.17 | -0.05 | 0.39 | 0.132 | 0.508 |
| TS | DBIL | 0.07 | 0.05 | 0.08 | 3.4E-23 |  | 0.07 | -0.01 | 0.14 | 0.082 | 0.916 |
| Ferritin | DBIL | 0.19 | 0.14 | 0.24 | 9.5E-16 |  | 0.17 | -0.04 | 0.38 | 0.119 | 0.624 |
| Transferrin | DBIL | -0.10 | -0.11 | -0.08 | 2.1E-28 |  | -0.06 | -0.12 | 0.00 | 0.040 | 0.093 |
| Serum Iron | TBIL | 0.63 | 0.52 | 0.74 | 1.5E-29 |  | 0.80 | -0.09 | 1.68 | 0.077 | 0.700 |
| TS | TBIL | 0.42 | 0.36 | 0.49 | 1.9E-38 |  | 0.32 | 0.02 | 0.62 | 0.039 | 0.337 |
| Ferritin | TBIL | 1.26 | 1.01 | 1.52 | 5.7E-23 |  | 0.80 | -0.06 | 1.65 | 0.067 | 0.142 |
| Transferrin | TBIL | -0.56 | -0.65 | -0.48 | 8.0E-39 |  | -0.29 | -0.52 | -0.06 | 0.013 | 2.6E-04 |

ALP, alkaline phosphatase; ALT, alanine aminotransferase; AST, aspartate aminotransferase; GGT, gamma glutamyltransferase; DBIL, direct bilirubin; TBIL, total bilirubin; TS, transferrin saturation; MR, Mendelian randomization; 95% CI, 95% confidence interval.

^1^ Beta represented the MR effect which were of standardized units for liver biomarkers.

**Table S6**. Associations between genetically predicted systemic iron status and liver diseases using weighted median and MR-Egger method.

| **Exposure** | **Outcome** | **Weighted median** | | | |  | **MR-Egger** | | | | |
| --- | --- | --- | --- | --- | --- | --- | --- | --- | --- | --- | --- |
|  |  | **OR** | **95% CI** | | ***P*-value** |  | **OR** | **95% CI** | | ***P*-value** | **Intercept *P*-value** |
| Serum Iron | NAFLD | 1.70 | 0.80 | 3.60 | 0.164 |  | 15.19 | 1.45 | 159.42 | 0.023 | 0.073 |
| TS | NAFLD | 1.73 | 1.09 | 2.72 | 0.019 |  | 2.79 | 1.13 | 6.90 | 0.026 | 0.221 |
| Ferritin | NAFLD | 5.93 | 1.49 | 23.63 | 0.012 |  | 14.62 | 1.43 | 149.82 | 0.024 | 0.315 |
| Transferrin | NAFLD | 0.43 | 0.23 | 0.80 | 0.008 |  | 0.41 | 0.18 | 0.92 | 0.030 | 0.830 |
| Serum Iron | ALD | 1.96 | 0.97 | 3.99 | 0.062 |  | 12.55 | 0.38 | 412.42 | 0.156 | 0.254 |
| TS | ALD | 1.84 | 1.18 | 2.88 | 0.007 |  | 2.53 | 0.59 | 10.89 | 0.214 | 0.484 |
| Ferritin | ALD | 14.62 | 1.43 | 149.82 | 0.005 |  | 11.90 | 0.35 | 407.11 | 0.169 | 0.520 |
| Transferrin | ALD | 0.48 | 0.26 | 0.87 | 0.016 |  | 0.46 | 0.11 | 1.89 | 0.280 | 0.913 |
| Serum Iron | Fibrosis/ Cirrhosis | 1.34 | 0.51 | 3.52 | 0.549 |  | 26.77 | 1.91 | 375.98 | 0.015 | 0.020 |
| TS | Fibrosis/ Cirrhosis | 1.60 | 0.93 | 2.73 | 0.088 |  | 3.65 | 1.35 | 9.88 | 0.011 | 0.028 |
| Ferritin | Fibrosis/ Cirrhosis | 4.79 | 0.98 | 23.44 | 0.053 |  | 26.42 | 2.03 | 344.06 | 0.012 | 0.043 |
| Transferrin | Fibrosis/ Cirrhosis | 0.45 | 0.23 | 0.90 | 0.025 |  | 0.31 | 0.12 | 0.75 | 0.009 | 0.107 |
| Serum Iron | Viral Hepatitis | 1.01 | 0.50 | 2.03 | 0.981 |  | 0.99 | 0.08 | 12.05 | 0.993 | 0.975 |
| TS | Viral Hepatitis | 1.00 | 0.62 | 1.61 | 0.986 |  | 0.98 | 0.37 | 2.56 | 0.963 | 0.973 |
| Ferritin | Viral Hepatitis | 0.99 | 0.23 | 4.21 | 0.988 |  | 0.98 | 0.08 | 11.62 | 0.986 | 0.941 |
| Transferrin | Viral Hepatitis | 1.06 | 0.54 | 2.05 | 0.872 |  | 1.04 | 0.44 | 2.46 | 0.930 | 0.948 |
| Serum Iron | Malignant neoplasm | 1.66 | 0.67 | 4.12 | 0.270 |  | 19.66 | 1.28 | 301.12 | 0.032 | 0.071 |
| TS | Malignant neoplasm | 1.80 | 1.05 | 3.08 | 0.033 |  | 3.20 | 1.12 | 9.15 | 0.030 | 0.153 |
| Ferritin | Malignant neoplasm | 6.70 | 1.32 | 33.98 | 0.022 |  | 19.33 | 1.30 | 288.03 | 0.032 | 0.227 |
| Transferrin | Malignant neoplasm | 0.41 | 0.20 | 0.84 | 0.015 |  | 0.35 | 0.14 | 0.89 | 0.027 | 0.521 |

NAFLD, nonalcoholic fatty liver disease; ALD, Alcoholic liver disease; TS, transferrin saturation; MR, Mendelian randomization; OR, odds ratio; 95% CI, 95% confidence interval.

**Table S7**. Sex-specific associations of genetically predicted iron status and liver diseases using weighted median and MR-Egger method.

| **Exposure** | | **Outcome** | **Weighted median** | | | |  | **MR-Egger** | | | | |  |  |
| --- | --- | --- | --- | --- | --- | --- | --- | --- | --- | --- | --- | --- | --- | --- |
|  |  |  | **OR** | **95% CI** | | ***P*-value** |  | **OR** | **95% CI** | | ***P*-value** | **Intercept *P*-value** |  |  |
| Women | |  |  |  |  |  |  |  |  |  |  |  |  |  |
|  | Serum Iron | NAFLD | 1.82 | 0.65 | 5.07 | 0.253 |  | 7.22 | 0.17 | 307.70 | 0.302 | 0.400 |  |  |
|  | TS | NAFLD | 1.68 | 0.85 | 3.31 | 0.136 |  | 2.04 | 0.44 | 9.45 | 0.363 | 0.598 |  |  |
|  | Ferritin | NAFLD | 4.79 | 0.62 | 36.92 | 0.132 |  | 6.87 | 0.16 | 299.01 | 0.317 | 0.622 |  |  |
|  | Transferrin | NAFLD | 0.58 | 0.23 | 1.46 | 0.245 |  | 0.56 | 0.13 | 2.39 | 0.430 | 0.925 |  |  |
| Men | |  |  |  |  |  |  |  |  |  |  |  |  | |
|  | Serum Iron | NAFLD | 1.74 | 0.63 | 4.77 | 0.285 |  | 29.34 | 1.20 | 718.18 | 0.038 | 0.105 |  |  |
|  | TS | NAFLD | 2.00 | 1.08 | 3.70 | 0.027 |  | 3.69 | 1.08 | 12.62 | 0.037 | 0.261 |  |  |
|  | Ferritin | NAFLD | 9.76 | 1.50 | 63.66 | 0.017 |  | 28.51 | 1.20 | 675.63 | 0.038 | 0.377 |  |  |
|  | Transferrin | NAFLD | 0.33 | 0.14 | 0.77 | 0.011 |  | 0.31 | 0.10 | 0.93 | 0.037 | 0.841 |  |  |
| Women | |  |  |  |  |  |  |  |  |  |  |  |  |  |
|  | Serum Iron | Fibrosis/ Cirrhosis | 0.86 | 0.31 | 2.38 | 0.766 |  | 1.92 | 0.06 | 65.25 | 0.716 | 0.607 |  |  |
|  | TS | Fibrosis/ Cirrhosis | 0.96 | 0.48 | 1.93 | 0.920 |  | 1.33 | 0.34 | 5.17 | 0.678 | 0.504 |  |  |
|  | Ferritin | Fibrosis/ Cirrhosis | 0.90 | 0.11 | 7.11 | 0.918 |  | 1.95 | 0.06 | 63.98 | 0.707 | 0.503 |  |  |
|  | Transferrin | Fibrosis/ Cirrhosis | 0.96 | 0.38 | 2.45 | 0.938 |  | 0.75 | 0.22 | 2.52 | 0.639 | 0.425 |  |  |
| Men | |  |  |  |  |  |  |  |  |  |  |  |  |  |
|  | Serum Iron | Fibrosis/ Cirrhosis | 1.44 | 0.34 | 6.15 | 0.619 |  | 387.19 | 8.61 | 17409.42 | 0.002 | 0.005 |  |  |
|  | TS | Fibrosis/ Cirrhosis | 2.31 | 1.05 | 5.06 | 0.037 |  | 10.14 | 2.35 | 43.79 | 0.002 | 0.017 |  |  |
|  | Ferritin | Fibrosis/ Cirrhosis | 17.92 | 1.75 | 183.79 | 0.015 |  | 371.17 | 8.58 | 16057.15 | 0.002 | 0.032 |  |  |
|  | Transferrin | Fibrosis/ Cirrhosis | 0.22 | 0.08 | 0.59 | 0.003 |  | 0.12 | 0.03 | 0.46 | 0.002 | 0.143 |  |  |
| Women | |  |  |  |  |  |  |  |  |  |  |  |  |  |
|  | Serum Iron | Viral Hepatitis | 0.67 | 0.22 | 2.02 | 0.482 |  | 0.15 | 0.00 | 7.19 | 0.339 | 0.463 |  |  |
|  | TS | Viral Hepatitis | 0.65 | 0.31 | 1.36 | 0.254 |  | 0.48 | 0.11 | 2.12 | 0.334 | 0.620 |  |  |
|  | Ferritin | Viral Hepatitis | 0.25 | 0.03 | 2.32 | 0.224 |  | 0.15 | 0.00 | 7.02 | 0.337 | 0.706 |  |  |
|  | Transferrin | Viral Hepatitis | 1.90 | 0.68 | 5.27 | 0.219 |  | 1.93 | 0.51 | 7.31 | 0.331 | 0.956 |  |  |
| Men | |  |  |  |  |  |  |  |  |  |  |  |  |  |
|  | Serum Iron | Viral Hepatitis | 1.42 | 0.55 | 3.63 | 0.469 |  | 3.86 | 0.14 | 103.40 | 0.420 | 0.504 |  |  |
|  | TS | Viral Hepatitis | 1.37 | 0.73 | 2.58 | 0.327 |  | 1.64 | 0.46 | 5.79 | 0.445 | 0.642 |  |  |
|  | Ferritin | Viral Hepatitis | 2.69 | 0.40 | 18.18 | 0.309 |  | 3.75 | 0.14 | 97.01 | 0.426 | 0.677 |  |  |
|  | Transferrin | Viral Hepatitis | 0.69 | 0.29 | 1.65 | 0.402 |  | 0.66 | 0.21 | 2.06 | 0.476 | 0.899 |  |  |

NAFLD, nonalcoholic fatty liver disease; ALD, Alcoholic liver disease; TS, transferrin saturation; MR, Mendelian randomization; OR, odds ratio; 95% CI, 95% confidence interval.

**Table S8**. Statistical power for sex-specific analyses.

| **Exposure** | **Women** | | |  | **Men** | | |
| --- | --- | --- | --- | --- | --- | --- | --- |
|  | **NAFLD** | **Fibrosis/ Cirrhosis** | **Viral Hepatitis** |  | **NAFLD** | **Fibrosis/ Cirrhosis** | **Viral Hepatitis** |
| Serum Iron | 21% | 8% | 12% |  | 86% | 67% | 11% |
| TS | 24% | 7% | 15% |  | 90% | 91% | 13% |
| Ferritin | 10% | 6% | 10% |  | 100% | 100% | 21% |
| Transferrin | 14% | <5% | 37% |  | 32% | 35% | 10% |

NAFLD, nonalcoholic fatty liver disease; TS, transferrin saturation.

Statistical power was calculated with mRnd (https://shiny.cnsgenomics.com/mRnd/) as a function of the sample size and the variance explained per instrumental variable for specific exposure and outcome, as well as odds ratios.

**Table S9**. Weighted median and MR-Egger estimators of genetically predicted systemic iron status with NAFLD in the replication analysis.

| **Exposure** |  | **Weighted median** | | | |  | **MR-Egger** | | | | |
| --- | --- | --- | --- | --- | --- | --- | --- | --- | --- | --- | --- |
|  |  | **OR** | **95% CI** | | ***P*-value** |  | **OR** | **95% CI** | | ***P*-value** | **Intercept *P*-value** |
| Serum Iron |  | 1.13 | 0.98 | 1.31 | 0.091 |  | 1.80 | 1.05 | 3.08 | 0.033 | 0.123 |
| Transferrin Saturation |  | 1.15 | 1.04 | 1.27 | 0.007 |  | 1.25 | 1.02 | 1.53 | 0.035 | 0.394 |
| Ferritin |  | 1.57 | 1.16 | 2.14 | 0.004 |  | 1.78 | 1.05 | 3.04 | 0.034 | 0.580 |
| Transferrin |  | 0.81 | 0.70 | 0.93 | 0.003 |  | 0.82 | 0.68 | 0.99 | 0.039 | 0.644 |

MR, Mendelian randomization; NAFLD, nonalcoholic fatty liver disease; OR, odds ratio; 95% CI, 95% confidence interval.

**Table S10**. Associations of genetically predicted NAFLD with systemic iron status in reverse MR analyses.

| **Outcome** | **Method** | **OR** | **95% CI** | | ***P*-value** | **Intercept *P*-value** |
| --- | --- | --- | --- | --- | --- | --- |
| Serum Iron | IVW | 1.01 | 0.98 | 1.04 | 0.577 |  |
| Serum Iron | Weighted median | 1.01 | 0.97 | 1.04 | 0.673 |  |
| Serum Iron | MR-Egger | 1.01 | 0.92 | 1.11 | 0.828 | 0.946 |
| Transferrin Saturation | IVW | 1.02 | 0.97 | 1.07 | 0.436 |  |
| Transferrin Saturation | Weighted median | 1.03 | 0.99 | 1.06 | 0.149 |  |
| Transferrin Saturation | MR-Egger | 1.08 | 0.93 | 1.25 | 0.393 | 0.487 |
| Ferritin | IVW | 1.04 | 1.01 | 1.07 | 0.005 |  |
| Ferritin | Weighted median | 1.03 | 1.00 | 1.07 | 0.064 |  |
| Ferritin | MR-Egger | 1.02 | 0.95 | 1.11 | 0.593 | 0.733 |
| Transferrin | IVW | 0.98 | 0.92 | 1.04 | 0.520 |  |
| Transferrin | Weighted median | 0.97 | 0.93 | 1.01 | 0.132 |  |
| Transferrin | MR-Egger | 0.86 | 0.75 | 0.98 | 0.113 | 0.133 |

IVW, inverse variance weighted; MR, Mendelian randomization; NAFLD, nonalcoholic fatty liver disease; OR, odds ratio; 95% CI, 95% confidence interval.
